# Elder abuse: the hidden face of domestic violence

**DOI:** 10.1080/07853890.2021.1896175

**Published:** 2021-09-28

**Authors:** Iris Almeida, Ana Filipa Carreiro, Ana Filipa Fernandes, Catarina Frade, Carolina Nobre, Lúcia Osório, Margarida Pereira, Ricardo Ventura Baúto

**Affiliations:** a Instituto Universitário Egas Moniz (IUEM), Egas Moniz Cooperativa de Ensino Superior, Caparica, Portugal; b Centro de Investigação Interdisciplinar Egas Moniz (CiiEM), Egas Moniz Cooperativa de Ensino Superior, Caparica, Portugal; c Laboratório de Psicologia Egas Moniz (LabPSI-EM), Centro de Investigação Interdisciplinar Egas Moniz (CiiEM), Egas Moniz Cooperativa de Ensino Superior, Caparica, Portugal; d Laboratório de Ciências Forenses e Psicológicas Egas Moniz (LCFPEM), Centro de Investigação Interdisciplinar Egas Moniz (CiiEM), Egas Moniz Cooperativa de Ensino Superior, Caparica, Portugal; e Gabinete de Informação e Atendimento à Vítima – Espaço Cidadania e Justiça (GIAV – Egas Moniz/DIAP Lisboa), Lisboa, Portugal

## Abstract

**Introduction:**

Violence against the elderly constitutes an undeniable and serious violation of human rights and affects the physical and psychological integrity of the victim. Is not a new phenomenon, is a worldwide problem that has become more pronounce in contemporary societies because of the ageing of the population. World Health Organization [1] defines elder violence as a single or repeated action, or the absence of an appropriate action, arising in the context of a relationship where there is an expectation of trust that causes suffering or harm to an elderly person. Occurs through several behaviours involving psychological, physical, sexual, financial violence, neglect and self-neglect [2]. The purpose of this paper is to demonstrate the work developed by the Victims Information and Assistance Office (GIAV) and by Forensic Psychology Office (GPF) at Egas Moniz Higher Education School about elder abuse.

**Materials and methods:**

The sample (*n* = 14) is derived from the domestic violence risk assessments of GIAV and GPF. We assessed 6 victims: 2 women and 4 man, aged between 64 and 95 years old (*M* = 76.67, *sd* = 10.71); and 8 defendants: 6 women and 2 man, aged between 24 and 77 years old (*M* = 46.13, *sd* = 15.52). The relationship between victims and defendants are 13 sons/daughters and 1 tenant. Data were collected from lawsuits, semi-structured interviews of the victims and defendants, collateral information and criminal record. All ethical issues have been taken due to the sensitive nature of the involved data involved and the respective informed consentient which contained the purpose of the assesses, the confidentiality limits, and information about the ethics and technician’s impartiality was sign by all participants.

**Results:**

The results demonstrated physical and psychological abuse (in all cases), followed by economical abuse (*n* = 13, 92.9%) and social abuse (*n* = 3, 21.4%). It is possible to identify several victims’ risk factors, namely gender (female victims – *n* = 11, 78.6%), physical problems/limitations (*n* = 11, 78.6%), age above 75 years old (*n* = 8, 57.1%) and previous abuse (*n* = 6, 42.9%). The most relevant offender’s risk factors are financial problems (*n* = 12, 85.7%), deficit in the coping skills (*n* = 12, 85.7%), others blame (*n* = 10, 71.4%), history of violence against others (*n* = 8, 57.1%), aggressiveness (*n* = 8, 57.1%), criminal history (*n* = 6, 42.9%), victim of domestic violence in the past (*n* = 8, 35.7%) and perpetrator of domestic violence in the past (*n* = 5, 35.7%). Finally external/relational factors are: offender’s dependence (*n* = 11, 78.6%), cohabitation (*n* = 11, 78.6%), history of conflicts between victim and offender (*n* = 10, 71.4%), poor emotional attachment or low family cohesion (*n* = 10, 71.4%), social isolation or lack of social support (*n* = 8, 57.1%), intergenerational transmission of violence (*n* = 7, 50%), inability in the performance of caregiver tasks (*n* = 5, 35.7%) and inexperience as caregiver (*n* = 5, 35.7%).

**Discussion and conclusions:**

Portugal it’s one of the top five European Countries with higher percentage (39%) of elderly mistreated [3], however, elder abuse is still the hidden face of domestic violence. The data show several risk factors for elder abuse. These results demonstrated the urgency about elder abuse risk assessment in criminal justice system and the need of a good articulation between Forensic Psychology and Law in order to demystify the hidden face of elder abuse.
